# Supplementary material for: Time in Range for Closed-Loop Systems versus Standard of Care during Physical Exercise in People with Type 1 Diabetes: A Systematic Review and Meta-Analysis
Source: J Clin Med. 2021 May 31;10(11):2445. doi: 10.3390/jcm10112445 (PMC8198013; doi:10.3390/jcm10112445)
Supplement: Supplementary file 1 [file jcm-10-02445-s001.zip › jcm-1214094-supplementary/JCM_Supplemental_Material_Eckstein.pdf]

Supplemental Material

Author	CLS N	CLS Mean	CLS SD	Control N	Control Mean	Control SD	AMD	95% CI	Weight
**Adolescents**									
Elleri D (a)	12	77.75	12.98	12	51.50	13.26	26.25	[15.75; 36.75]	7.3%
Elleri D (b)	12	65.50	9.83	12	63.75	22.21	1.75	[−11.99; 15.49]	5.5%
Dovc K (a)	20	79.58	6.97	20	69.72	6.12	9.85	[5.78; 13.92]	12.0%
Dovc K (b)	20	77.53	6.58	20	66.53	6.28	11.00	[7.02; 14.98]	12.1%
Breton M	32	63.20	31.10	32	62.80	31.40	0.40	[−14.91; 15.71]	4.8%
Ekhlaspour L	48	57.80	27.30	48	55.90	31.10	1.90	[−9.81; 13.61]	6.6%
**Overall effect**	**144**			**144**			**9.46**	**[2.48; 16.45]**	**48.4%**
Heterogeneity: $I^2 = 65\%$, $\tau^2 = 51.43$, $p = 0.01$									
**Adults**									
Jacobs P (a)	21	67.25	3.75	21	68.00	4.00	−0.75	[−3.10; 1.60]	13.0%
Jacobs P (b)	21	72.00	3.00	21	70.50	1.50	1.50	[0.07; 2.93]	13.4%
Castle J (a)	20	84.30	16.70	20	78.20	26.20	6.10	[−7.52; 19.72]	5.6%
Castle J (b)	20	83.30	16.70	20	78.20	26.20	5.10	[−8.52; 18.72]	5.6%
Castle J (c)	20	84.30	16.70	20	78.30	18.90	6.00	[−5.05; 17.05]	7.0%
Castle J (d)	20	83.30	16.70	20	78.30	18.90	5.00	[−6.05; 16.05]	7.0%
**Overall effect**	**122**			**122**			**1.07**	**[−0.81; 2.96]**	**51.6%**
Heterogeneity: $I^2 = 0\%$, $\tau^2 = 1.15$, $p = 0.44$									
**Overall effect**	**266**			**266**			**6.18**	**[1.99; 10.38]**	**100.0%**

Heterogeneity: $I^2 = 82\%$, $\tau^2 = 33.68$, $p < 0.01$
Residual heterogeneity: $I^2 = 47\%$, $p = 0.04$

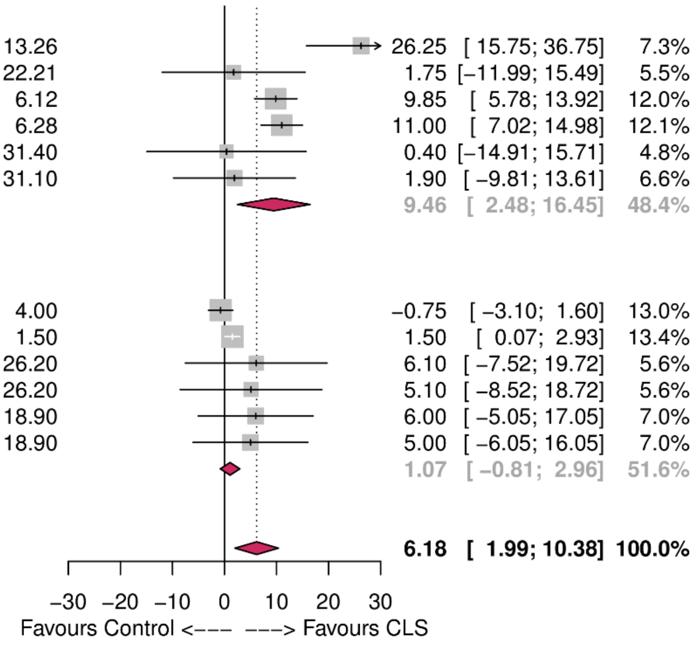

Figure S1: Subgroup analysis on age

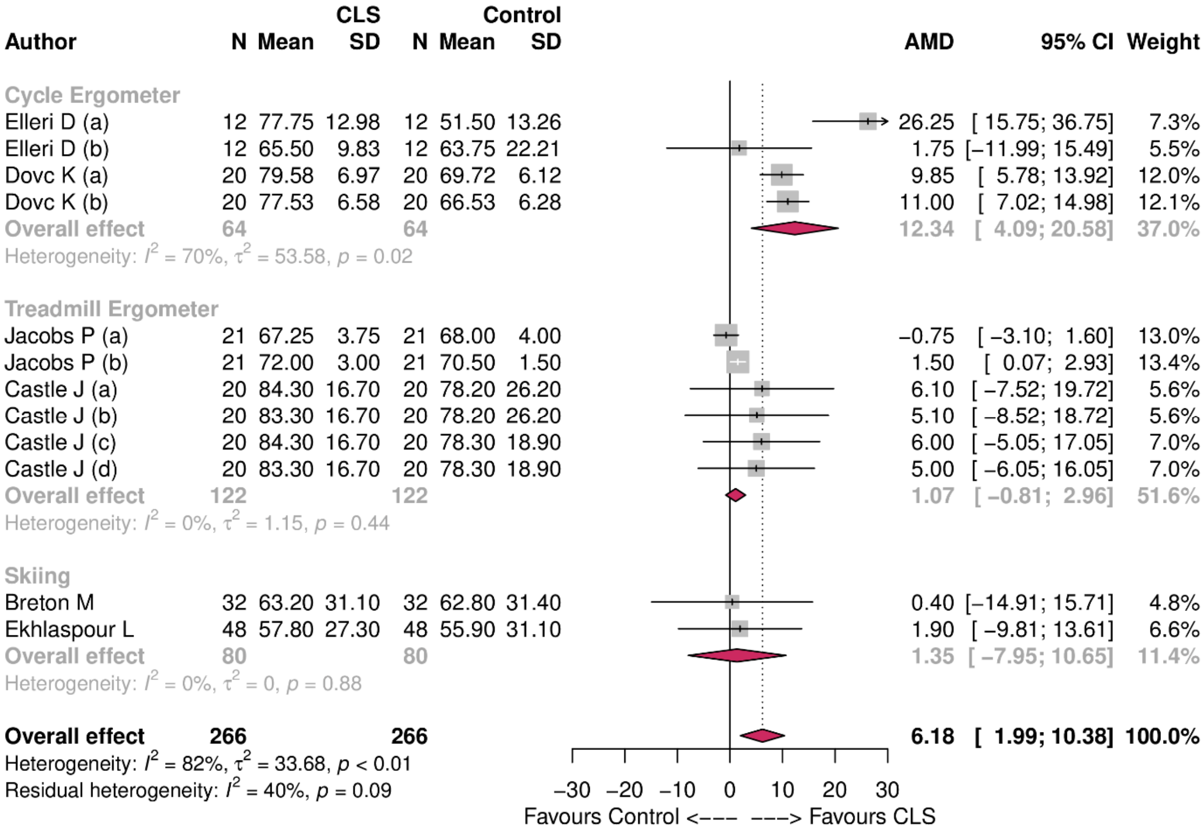

Figure S2: Subgroup analysis on type of exercise

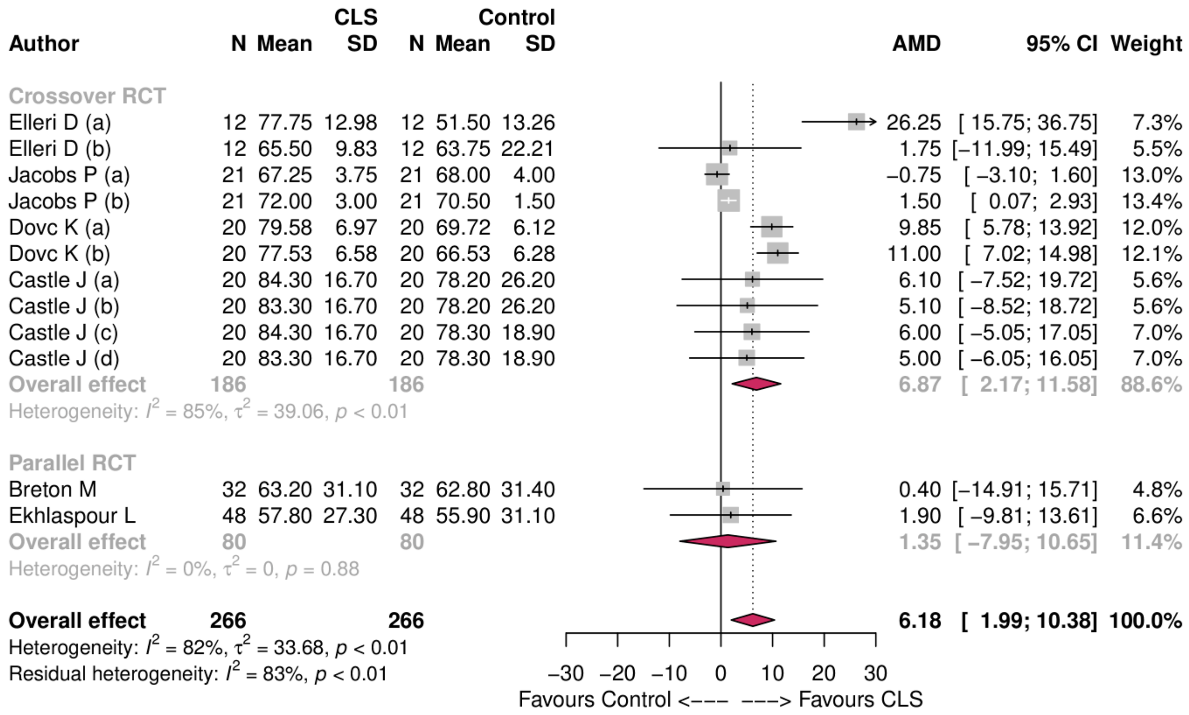

Figure S3: Subgroup analysis on study type

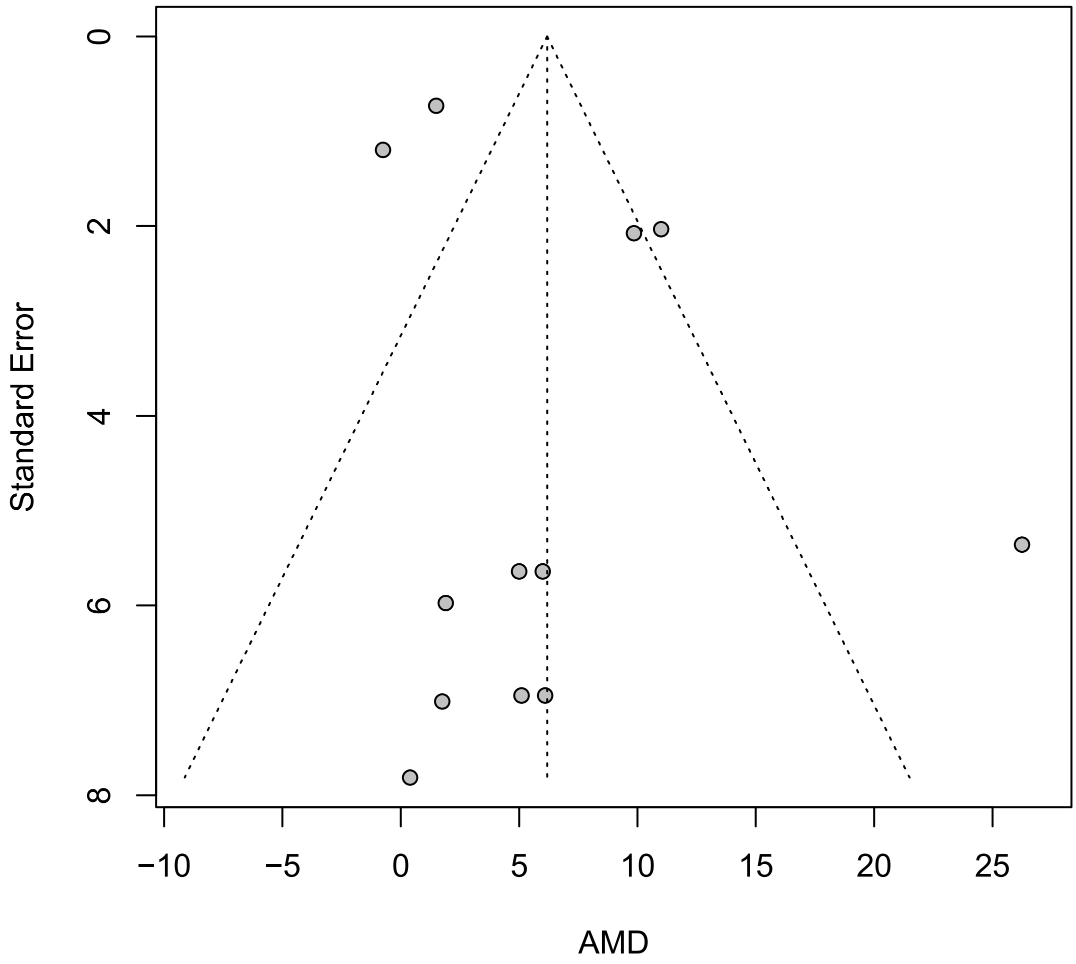

Figure S4: *Egger's test: p-value = 0.13

Table S1: Search Pubmed, January 2020

#	Term	Hits
#1	(diabetes mellitus, type 1 [mh])	74437
#2	(type 1 diabetes mellitus [tw])	77016
#3	(type 1 diabetes [tw])	38803
#4	("type i" diabetes mellitus [tw])	8600
#5	("type-i" diabetes mellitus [tw])	8600
#6	("insulin-dependent" diabet* [tw])	27696
#7	(Artificial pancreas [mh])	735
#8	(Bioartificial Organs [mh] AND (pancreas [tw] OR insulin [tw] OR diabet* [tw]))	75
#9	(Bionics [mh] AND (pancreas [tw] OR insulin [tw] OR diabet* [tw]))	8
#10	("synthetic pancreas" [tw] AND (insulin [tw] OR diabet* [tw]))	4
#11	("artificial endocrine pancreas" [tw] AND (insulin [tw] OR diabet* [tw]))	195
#12	(artificial beta cell* [tw] OR artificial b cell* [tw] OR artificial b-cell* [tw])	159
#13	(closed-loop* [tw] AND (pancreas [tw] OR insulin [tw] OR diabet* [tw]))	1124
#14	("closed loop*" AND (pancreas [tw] OR insulin [tw] OR diabet* [tw]))	1125
#15	("bioartificial pancreas" [tw] OR "bio-artificial pancreas" [tw])	269
#16	#1 - #15 OR	104067
#17	(insulin pump [tw])	2184
#18	(insulin delivery system* [tw])	488
#19	(insulin [tw])	406540
#20	(Infusion Pumps, Implantable [mh])	3616
#21	(Insulin Infusion System [mh])	5136
#22	(continuous subcutaneous insulin infusion [tw])	1935
#23	(csii [tw])	1492
#24	#17 - #23 OR	409716
#25	#16 AND #24	56261
#26	(glucose [tw] AND (sensor* [tw] OR sensing*))	13781
#27	("sensed glucose" [tw])	8

#28	(CGM [tw])	2135
#29	(CGMS [tw])	642
#30	(RTCGM [tw])	39
#31	(RTCGMS [tw])	3
#32	(ICGM [tw])	11
#33	(glucoWatch [tw])	54
#34	(medtronic [tw] AND diabet*)	423
#35	(dexcom [tw] AND diabet*)	154
#36	(abbott [tw] AND diabet*)	298
#37	(omnipod [tw] AND diabet*)	37
#38	(tandem [tw] AND diabet*)	2472
#39	(animas [tw] AND diabet*)	17
#40	(roche [tw] AND diabet*)	361
#41	#26 – #40 OR	18984
#42	#16 AND #24 AND #41	1943
#43	(Exercise [mh])	187953
#44	(Resistance Training [mh])	8109
#45	(High-Intensity Interval Training [mh])	850
#46	(high-intensity training [tw])	703
#47	(high intensity training [tw])	703
#48	(physical activity [tw])	104733
#49	#43 – #48 OR	253679
#50	#16 AND #24 AND #41 AND #49	92
#51	Limit: humans and english language	79

Table S2: Search Embase, January 2020

#	Term	Hits
#1	(diabetes mellitus, type 1).mp.	3186
#2	(type 1 diabetes mellitus).mp.	16025
#3	(type 1 diabetes).mp.	62482
#4	("type i" diabetes mellitus).mp.	2474
#5	("type-i" diabetes mellitus).mp.	2474
#6	("insulin-dependent" diabet*).mp.	340700
#7	(artificial pancreas).mp.	2906
#8	(Bioartificial Organs AND (pancreas OR insulin OR diabet*)).mp.	22
#9	(Bionics AND (pancreas OR insulin OR diabet*)).mp.	27
#10	("synthetic pancreas" AND (insulin OR diabet*)).mp.	7
#11	("artificial endocrine pancreas" AND (insulin OR diabet*)).mp.	276
#12	(artificial beta cell* OR artificial b cell* OR artificial b-cell*).mp.	210
#13	(closed-loop* AND (pancreas OR insulin OR diabet*)).mp.	2072
#14	("closed loop*" AND (pancreas OR insulin OR diabet*)).mp.	2072
#15	("bioartificial pancreas" OR "bio-artificial pancreas").mp.	391
#16	#1 - #15 OR	352561
#17	(insulin pump).mp.	7940
#18	(insulin delivery system*).mp.	759
#19	(insulin).mp.	765097
#20	(Infusion Pumps, Implantable).mp.	15
#21	(Insulin Infusion System).mp.	187
#22	(continuous subcutaneous insulin infusion).mp.	3371
#23	(csii).mp.	3418
#24	#17 - #23 OR	765224
#25	#16 AND #24	346428
#26	(glucose AND (sensor* OR sensing*)).mp.	22379
#27	("sensed glucose").mp.	11

#28	(CGM).mp.	4836
#29	(CGMS).mp.	1501
#30	(RTCGM).mp.	104
#31	(RTCGMS).mp.	9
#32	(ICGM).mp.	30
#33	(glucoWatch).mp.	193
#34	(medtronic AND diabet*).mp.	3001
#35	(dexcom AND diabet*).mp.	812
#36	(abbott AND diabet*).mp.	2774
#37	(omnipod AND diabet*).mp.	208
#38	(tandem AND diabet*).mp.	3323
#39	(animas AND diabet*).mp.	219
#40	(roche AND diabet*).mp.	3671
#41	#26 – #40 OR	37013
#42	#16 AND #24 AND #41	10448
#43	(Exercise).mp.	479276
#44	(Resistance Training).mp.	20024
#45	(High-Intensity Interval Training).mp.	2549
#46	(high-intensity training).mp.	860
#47	(high intensity training).mp.	860
#48	(physical activity).mp.	201470
#49	#43 – #48 OR	618491
#50	#16 AND #24 AND #41 AND #49	956
#51	Limit: human and english language	882

Table S3: Search ISY Web of Science, January 2020

#	Term	Hits
#1	TS= (diabetes mellitus, type 1)	220703
#2	TS= (type 1 diabetes mellitus)	220703
#3	TS= (type 1 diabetes)	250449
#4	TS= ("type i" diabetes mellitus)	17553
#5	TS= ("type-i" diabetes mellitus)	17553
#6	TS= ("insulin-dependent" diabet*)	167753
#7	TS= (artificial pancreas)	5821
#8	TS= (Bioartificial Organs AND (pancreas OR insulin OR diabet*))	298
#9	TS= (Bionics AND (pancreas OR insulin OR diabet*))	148
#10	TS= ("synthetic pancreas" AND (insulin OR diabet*))	4
#11	TS= ("artificial endocrine pancreas" AND (insulin OR diabet*))	328
#12	TS= (artificial beta cell* OR artificial b cell* OR artificial b-cell*)	28435
#13	TS= (closed-loop* AND (pancreas OR insulin OR diabet*))	1731
#14	TS= ("closed loop*" AND (pancreas OR insulin OR diabet*))	1722
#15	TS= ("bioartificial pancreas" OR "bio-artificial pancreas")	652
#16	#1 - #15 OR	353226
#17	TS= (insulin pump)	9641
#18	TS= (insulin delivery system*)	15831
#19	TS= (insulin)	738676
#20	TS= (Infusion Pumps, Implantable)	4399
#21	TS= (Insulin Infusion System)	25341
#22	TS= (continuous subcutaneous insulin infusion)	3830
#23	TS= (csii)	2280
#24	#17 - #23 OR	742532
#25	#16 AND #24	229761
#26	TS= (glucose AND (sensor* OR sensing*))	43648
#27	TS= ("sensed glucose")	10
#28	TS= (CGM)	4319

#29	TS= (CGMS)	1076
#30	TS= (RTCGM)	58
#31	TS= (RTCGMS)	4
#32	TS= (ICGM)	21
#33	TS= (glucoWatch)	119
#34	TS= (medtronic AND diabet*)	547
#35	TS= (dexcom AND diabet*)	229
#36	TS= (abbott AND diabet*)	381
#37	TS= (omnipod AND diabet*)	69
#38	TS= (tandem AND diabet*)	3395
#39	TS= (animas AND diabet*)	28
#40	TS= (roche AND diabet*)	553
#41	#26 – #40 OR	52534
#42	#16 AND #24 AND #41	6694
#43	TS= (Exercise)	711220
#44	TS= (Resistance Training)	43045
#45	TS= (High-Intensity Interval Training)	3993
#46	TS= (high-intensity training)	10682
#47	TS= (high intensity training)	23086
#48	TS= (physical activity)	694701
#49	#43 – #48 OR	1289571
#50	#16 AND #24 AND #41 AND #49	445
#51	#50 NOT (animal)	373
#52	Limit: english language	373

Table S4: Search Cochrane Controlled Register of Trials (CENTRAL)

#	Term	Hits
#1	(diabetes mellitus, type 1).mp.	5198
#2	(type 1 diabetes mellitus).mp.	1480
#3	(type 1 diabetes).mp.	5951
#4	("type i" diabetes mellitus).mp.	186
#5	("type-i" diabetes mellitus).mp.	186
#6	("insulin-dependent" diabet*).mp.	20400
#7	(artificial pancreas).mp.	274
#8	(Bioartificial Organs AND (pancreas OR insulin OR diabet*)).mp.	0
#9	(Bionics AND (pancreas OR insulin OR diabet*)).mp.	7
#10	("synthetic pancreas" AND (insulin OR diabet*)).mp.	0
#11	("artificial endocrine pancreas" AND (insulin OR diabet*)).mp.	21
#12	(artificial beta cell* OR artificial b cell* OR artificial b-cell*).mp.	6
#13	(closed-loop* AND (pancreas OR insulin OR diabet*)).mp.	474
#14	("closed loop*" AND (pancreas OR insulin OR diabet*)).mp.	474
#15	("bioartificial pancreas" OR "bio-artificial pancreas").mp.	0
#16	#1 - #15 OR	25261
#17	(insulin pump).mp.	1084
#18	(insulin delivery system*).mp.	95
#19	(insulin).mp.	57490
#20	(Infusion Pumps, Implantable).mp.	142
#21	(Insulin Infusion System).mp.	17
#22	(continuous subcutaneous insulin infusion).mp.	667
#23	(csii).mp.	672
#24	#17 - #23 OR	57598
#25	#16 AND #24	23374
#26	(glucose AND (sensor* OR sensing*)).mp.	1420
#27	("sensed glucose").mp.	1
#28	(CGM).mp.	1211

#29	(CGMS).mp.	308
#30	(RTCGM).mp.	24
#31	(RTCGMS).mp.	0
#32	(ICGM).mp.	1
#33	(glucoWatch).mp.	13
#34	(medtronic AND diabet*).mp.	283
#35	(dexcom AND diabet*).mp.	139
#36	(abbott AND diabet*).mp.	137
#37	(omnipod AND diabet*).mp.	13
#38	(tandem AND diabet*).mp.	174
#39	(animas AND diabet*).mp.	6
#40	(roche AND diabet*).mp.	133
#41	#26 – #40 OR	2987
#42	#16 AND #24 AND #41	1468
#43	(Exercise).mp.	86967
#44	(Resistance Training).mp.	8192
#45	(High-Intensity Interval Training).mp.	1435
#46	(high-intensity training).mp.	354
#47	(high intensity training).mp.	354
#48	(physical activity).mp.	27569
#49	#43 – #48 OR	102845
#50	#16 AND #24 AND #41 AND #49	209
#51	Limit: english language	140

Table S5: Secondary outcomes: time in percentage below and above target range during exercise and events <3.9 mmol/L

	**Time spent <3.9mmol/l during exercise**				**Time spent >10mmol/l during exercise**		**Number of events <3.9mmol/l during exercise:**	
	**Closed-loop single hormone**	**Closed-loop dual-hormone**	**PLGS**	**Current care**	**Closed-loop**	**Current care**	**Closed-loop**	**Current care**
Elleri D (3a)	n/a	n/a	n/a	n/a	n/a	n/a	n/a	n/a
Elleri D (3b)	n/a	n/a	n/a	n/a	n/a	n/a	n/a	n/a
Jacobs P (2a)	0.3 [-0.1, 0.7]	n/a	n/a	0.8 [0.1, 1.4]	32 [25–39]	31 [24–39]	n/a	n/a
Jacobs P (2b)	3.1 [0.8–5.3]	n/a	n/a	n/a	25 [19–30]	n/a	n/a	n/a
Dovc K (1a)	0.0 (0.0–0.0)	n/a	n/a	0.2 (0.0–4.5)	17.1 (7.2–33.0)	25.2 (6.0–39.3)	n/a	n/a
Dovc K (1b)	1.1 (0.0–3.4)	n/a	n/a	0.0 (0.0–3.3)	20.8 (5.0–28.9)	29.6 (17.6–45.4)	n/a	n/a
BretonM (5)	1.4 ± 1.6	n/a	n/a	1.4 ± 1.6	n/a	n/a	0.3 ± 0.4	0.3 ± 0.7
CastleJ (6)	3.4 ± 4.5	8.3 ± 12.6	7.6 ± 8.0	4.3 ± 6.8	n/a	n/a	n/a	n/a
Ekhlaspour L (4)	0 (0.0, 0.8)	n/a	n/a	0 (0.0, 0.4)	41.4 ± 27.8	41.5 ± 30.3	n/a	n/a

PLGS: predictive low-glucose suspend system

Table S6: Secondary outcomes: time in percentage during the post-exercise period including number of events <3.9 mmol/L

	**TIR during the post-exercise period:**		**Time spent <3.9mmol/l in the post-exercise period:**		**Time spent >10mmol/l during the post-exercise period:**		**Number of events <3.9mmol/l during the post-exercise:**	
	**Closed-loop**	**Current care**	**Closed-loop**	**Current care**	**Closed-loop**	**Current care**	**Closed-loop**	**Current care**
Elleri D (3a)	94 (54, 100)	79 (34, 100)	0.0 (0.0, 1.0)	0.0 (0.0, 2.4)	1.0 (0.0–13.9)	64.6 (1.6–97.5)	n/a	n/a
Elleri D (3b)	96 (79, 100)	33 (2, 80)	0.2 (0.0, 4.7)	0.0 (0.0, 0.0)	n/a	n/a	n/a	n/a
Jacobs P (2a)	n/a	n/a	n/a	n/a	n/a	n/a	n/a	n/a
Jacobs P (2b)	n/a	n/a	n/a	n/a	n/a	n/a	n/a	n/a
Dovc K (1a)	92.8 (69.8–98.4)	73.3 (61.3–84.2)	n/a	n/a	7.2 (0.2–23.9)	22.7 (9.1–38.7)	3 (in total)	4 (in total)
Dovc K (1b)	n/a	n/a	0.0 (0.0–3.5)	0.0 (0.0–2.8)	n/a	n/a	n/a	n/a
BretonM (5)	79.3 ± 29.8	68.8 ± 24.1	2.2 ± 2.3	2.5 ± 6.5	n/a	n/a	0.1 ± 0.3	0.1 ± 0.4
CastleJ (6)	n/a	n/a	n/a	n/a	n/a	n/a	n/a	n/a
Ekhlaspour L (4)	78.6 ± 20.3	50.9 ± 34.2	0 (0.0, 8.2)	0 (0.0, 6.4)	18.2 ± 21.4	44.5 ± 37	n/a	n/a
